# Impacts of CR1 genetic variants on cerebrospinal fluid and neuroimaging biomarkers in alzheimer’s disease

**DOI:** 10.1186/s12881-020-01114-x

**Published:** 2020-09-12

**Authors:** Xi-chen Zhu, Wen-zhuo Dai, Tao Ma

**Affiliations:** Department of Neurology, the Affiliated Wuxi No. 2 People’s Hospital of Nanjing Medical University, No. 68 Zhongshan Road, Wuxi, Jiangsu Province, 214002 China

**Keywords:** *CR1*, Alzheimer’s disease, Amyloid-β (Aβ) plaques, CSF Aβ_42_, ADNI

## Abstract

**Background:**

The complement component (3b/4b) receptor 1 gene (*CR1*) gene has been proved to affect the susceptibility of Alzheimer’s disease (AD) in different ethnic and districts groups. However, the effect of *CR1* genetic variants on amyloid β (Aβ) metabolism of AD human is still unclear. Hence, the aim of this study was to investigate genetic influences of *CR1* gene on Aβ metabolism.

**Methods:**

All data of AD patients and normal controls (NC) were obtained from alzheimer’s disease neuroimaging initiative database (ADNI) database. In order to assess the effect of each single nucleotide polymorphism (SNP) of *CR1* on Aβ metabolism, the PLINK software was used to conduct the quality control procedures to enroll appropriate SNPs. Moreover, the correlation between *CR1* genotypes and Aβ metabolism in all participants were estimated with multiple linear regression models.

**Results:**

After quality control procedures, a total of 329 samples and 83 SNPs were enrolled in our study. Moreover, our results identified five SNPs (rs10494884, rs11118322, rs1323721, rs17259045 and rs41308433), which were linked to Aβ accumulation in brain. In further analyses, rs17259045 was found to decrease Aβ accumulation among AD patients. Additionally, our study revealed the genetic variants in rs12567945 could increase CSF Aβ_42_ in NC population.

**Conclusions:**

Our study had revealed several novel SNPs in *CR1* genes which might be involved in the progression of AD via regulating Aβ accumulation. These findings will provide a new basis for the diagnosis and treatment AD.

## Highlights

1. We found that five SNPs were linked to Aβ accumulation in brain.
2. The rs17259045 decreased Aβ accumulation among AD patients.
3. The rs12567945 could increase CSF Aβ42 in NC population.

## Background

Alzheimer’s disease (AD) has been regarded as a neurodegenerative disease of the elderly, which has accounted for 47 million people worldwide with numbers predicted to rise double by 2030 and triple by 2050 [1]. As one of the most common dementia, AD has the characteristics of poor language, memory, perception, behavior and activities of daily living. Moreover, the extracellular neurotoxic amyloid-β (Aβ) plaques and intracellular neurofibrillary tangles have been regarded as the neuropathological hallmarks of AD [2]. It has been widely confirmed that AD is a multifactorial disease, and genetic factors is proved to play a vital role in AD [3, 4]. However, in spite of the progress in understanding risk factors related to AD development, the underlying mechanisms involved in this disease have not been completely understood till now, and to date there is no curative treatment for AD [5, 6].

Now many genes are proved to significantly influence AD risk, among which the complement component (3b/4b) receptor 1 gene (*CR1*) has been proved to affect AD susceptibility across different ethnic and districts groups [7–12]. Currently, CR1 has been postulated to be a key factor for AD pathogenesis because of its role in regulating complement activity by acting as a receptor of complement C3b protein [13]. More importantly, in AD patients, CR1 is found to be associated with neuronal death [14] and hence has received increasing attention. Although a significant association between AD and single nucleotide polymorphisms (SNPs) in several novel AD loci of large case-control datasets is identified, CR1 is considered as one of the most important genetic susceptibility loci in AD according to the Alzgene database [15–17]. As well known, accumulation of Aβ in brain is one important pathological hallmark of AD, moreover, it is considered to induce a deleterious neurodegenerative cascade and finally cause cognitive impairments [18]. Furthermore, it has been shown that *CR1* takes part in AD pathology by regulating the amyloid protein (Aβ) metabolism [19], and Johansson et al. [20] reveals that the single nucleotide polymorphisms (SNPs) in *CR1* gene were associated with increased erythrocyte *CR1* which will finally decreased AD risk. Hence, it would be meaningful to discover the genetic variants of *CR1* in AD development.

In this study, we enrolled the participants from the alzheimer’s disease neuroimaging initiative (ADNI) database (http://www.loni.ucla.edu/ADNI), which is a multicenter project to assess the role of genetic factors in neuroimage biomarkers and cerebrospinal fluid (CSF) proteins. Next, we used PLINK software to conduct the quality control procedures to enroll appropriate SNPs in *CR1*, and then investigated genetic influences of *CR1* gene on Aβ metabolism, in order to explore the role of *CR1* genetic variants in the progression of AD.

## Methods

### Participants

The data in our study were obtained from the ADNI database, which contains genetic information, neuroimaging information, and CSF proteins of AD, and normal controls (NC) (http:// www.adni-info.org). All participants of this study were included with the specific criteria according the protocol of ADNI, and then divided into two groups, including the AD group and NC group. Briefly, when participants met the National Institute of Neurological and Communicative Disorders (NINCDS) and Stroke/Alzheimer’s Disease and Related Disorders Association (ADRDA) criteria for probable AD [21], they were diagnosed as AD.

### Genotyping data

All genetic information of SNPs of *CR1* were detected using the Illumina Infinium Human610-Quad Bead Chip (Illumina, Inc., San Diego, CA) or Illumina Human Omni Express Bead Chip. And the quality control procedures were performed by using PLINK software. The SNPs would be excluded when minimum minor allele frequency (MAF) was less than 0.01 or Hardy-Weinberg (H-W) equilibrium test’s value was less than 0.05.

### AV45-pet

The imaging data of PET with amyloid tracer, florbetapir (AV-45), was obtained from UC Berkeley-AV45 analysis database [22]. In order to define cortical grey matter regions of interest, these images were segmented and parcellated with Freesurfer (Version 5.3.0). After that, four regions, including the frontal, cingulate, parietal, temporal and florbetapir were involved in this study [23]. In addition, through averaging across the four cortical regions and dividing it by whole cerebellum florbetapir, the cortical standardized uptake values ratios (SUVR) were calculated [24]..

### CSF Aβ_42_ proteins

Similarly, the data about the level of CSF Aβ_42_ was also got from ADNI database. Briefly, all samples of CSF were collected and transported to ADNI Biomarker Core laboratory at the University of Pennsylvania Medical Center. Following thawed at room temperature and gentle mixed, these samples were used for preparation of aliquots (0.5 ml). Finally, the level of CSF Aβ_42_ was determined with multiplex xMAP luminex platform (Luminex Corp, Austin, TX) with immunoassay kit according to reagents [25]_._

### Statistical analyses

All statistical analyses were determined by using the SPSS 18.0 software (SPSS Inc., Chicago, IL, USA) and PLINK (http:// pngu.mgh.harvard.edu/wpurcell/plink/). The demographic characteristics were performed with means ± standard deviations (SD). The t-test or chi-square test were used for the analysis of demographics and genotypic frequencies. The correlation between *CR1* genotypes and Aβ metabolism in all cohorts were estimated with multiple linear regression models. The false discovery rate (FDR) test was applied to control for multiple hypothesis testing [26], and a *P* ≤ 0.05 was considered to be statistically significant.

## Results

### Characteristics of included participants

As shown in Table 1, a total of 329 individuals (48 AD and 281 NC) were enrolled in our study according to the quality control for genotype. Moreover, the AD group with 70.8% has higher frequency of the ε4 allele within apolipoprotein E (ApoE) gene than the NC group with 26.3%. According to the scores of different neuropsychological scales, the patients with AD have worse cognitive function in comparison to those NC group, respectively.

**Table 1:** The details of enrolled samples from ADNI database

Characteristics	NC		AD	
	N	Mean ± SD		Mean ± SD
Age (years)	281	74.51 ± 5.56	48	75.51 ± 9.23
Gender (male/female)	281	136/145	48	30/18
Education (years)	281	16.41 ± 2.66	48	15.73 ± 2.62
ApoE ε4 (0/1/2)	281	204/70/7	48	14/25/9
CDRSB (scores)	207	6.54 ± 0.55	47	5.3 ± 0.72
ADAS (scores)	281	29.07 ± 1.15	48	22.96 ± 2.03
MMSE (scores)	281	9.06 ± 4.23	48	29.8 ± 8.44
RAVLT total (scores)	280	44.83 ± 9.6	47	22.32 ± 7.84
FAQ (scores)	281	0.17 ± 0.66	48	12.6 ± 7.14

*ADNI* alzheimer’s disease neuroimaging initiative, *AD* alzheimer’s disease, *ApoE ε4* apolipoprotein E ε4, *SD* standard deviations, *ADAS* alzheimer’s disease assessment scale, *CDRSB* clinical dementia rating scale sum of boxes, *FAQ* functional activities questionnaire, *MMSE* mini-mental state exam, *NC* normal controls, *RAVLT* rey auditory verbal learning test

### Characteristics of included SNPs of CR1

After quality control with PLINK software, a total of 83 SNPs of *CR1* were enrolled in our study. Next, we used Haploview version 4.2 to explore the linkage disequilibrium (LD) patterns of these included SNPs of *CR1* (**Supplementary Fig.** **1**). The results showed these SNPs distributed from block 1 to 5 which indicated the SNPs capture most common variants in *CR1*. Furthermore, the characteristics (major allele, minor allele, MAF, functional consequence, position and H-W value) of included *CR1* SNPs were illustrated in **supplementary Table** **1**. The MAF values of all included SNPs were more than 0.01, and the H-W values of included SNPs were more than 0.05.

### The effects of CR1 genetic variants on AV-45 PET

It is well known that the data of the AV-45 retention on the PET imaging of amyloid may represent Aβ accumulation biomarkers. In the present study, our data revealed five SNPs, including rs10494884, rs11118322, rs1323721, rs17259045 and rs41308433 were significantly related to the level of tracer retention on amyloid PET imaging. Moreover, Rs10494884, RS11118322, and rs1323721 were in block 3, rs17259045 was in block 2 and RS41308433 was in block 4. As illustrated in Table 2, the variant in rs10494884 would increase Aβ accumulation in temporal, frontal, and SUVR (*P* = 0.03392, *P* = 0.03845 and *P* = 0.04447). Similarly, rs11118322 and rs1323721 were proved to significantly increase Aβ accumulation in temporal and frontal (all, *P* < 0.05). In addition, our data revealed that the variant in rs17259045 may widely decrease the level of Aβ accumulation in frontal (*P* = 0.007581), temporal (*P* = 0.009251), SUVR (*P* = 0.01725), cingulated (*P* = 0.02512) and parietal (*P* = 0.03033). And rs41308433 was proved to reduce the Aβ accumulation only in temporal (*P* = 0.04292).

**Table 2:** The association of genetic variants in *CR1* gene with Aβ deposition on AV-45 PET among all people

SNPs	Gene regions	Position (Chromosome)	Major allele	Minor allele	Regions	β	***P*** value
rs10494884	intron variant	1:207674531	G	A	temporal	0.03364	0.03392
					frontal	0.03543	0.03845
					SUVR	0.02647	0.04447
rs11118322	intron variant	1:207674706	T	C	temporal	0.03342	0.03448
					frontal	0.03477	0.04155
rs1323721	intron variant	1:207649895	A	G	temporal	0.03245	0.04141
					frontal	0.03381	0.04894
rs17259045	missense	1:207609362	A	G	frontal	−0.0773	0.007581
					temporal	−0.06968	0.009251
					SUVR	−0.05298	0.01725
					cingulate	−0.07068	0.02512
					parietal	−0.06473	0.03033
rs41308433	intron variant	1:207699490	A	C	temporal	−0.0427	0.04292

*CR1* complement component (3b/4b) receptor 1 gene, *SNPs* single nucleotide polymorphisms, *Aβ* amyloid protein, *SUVR* standardized uptake values ratios

**Fig. 1:**
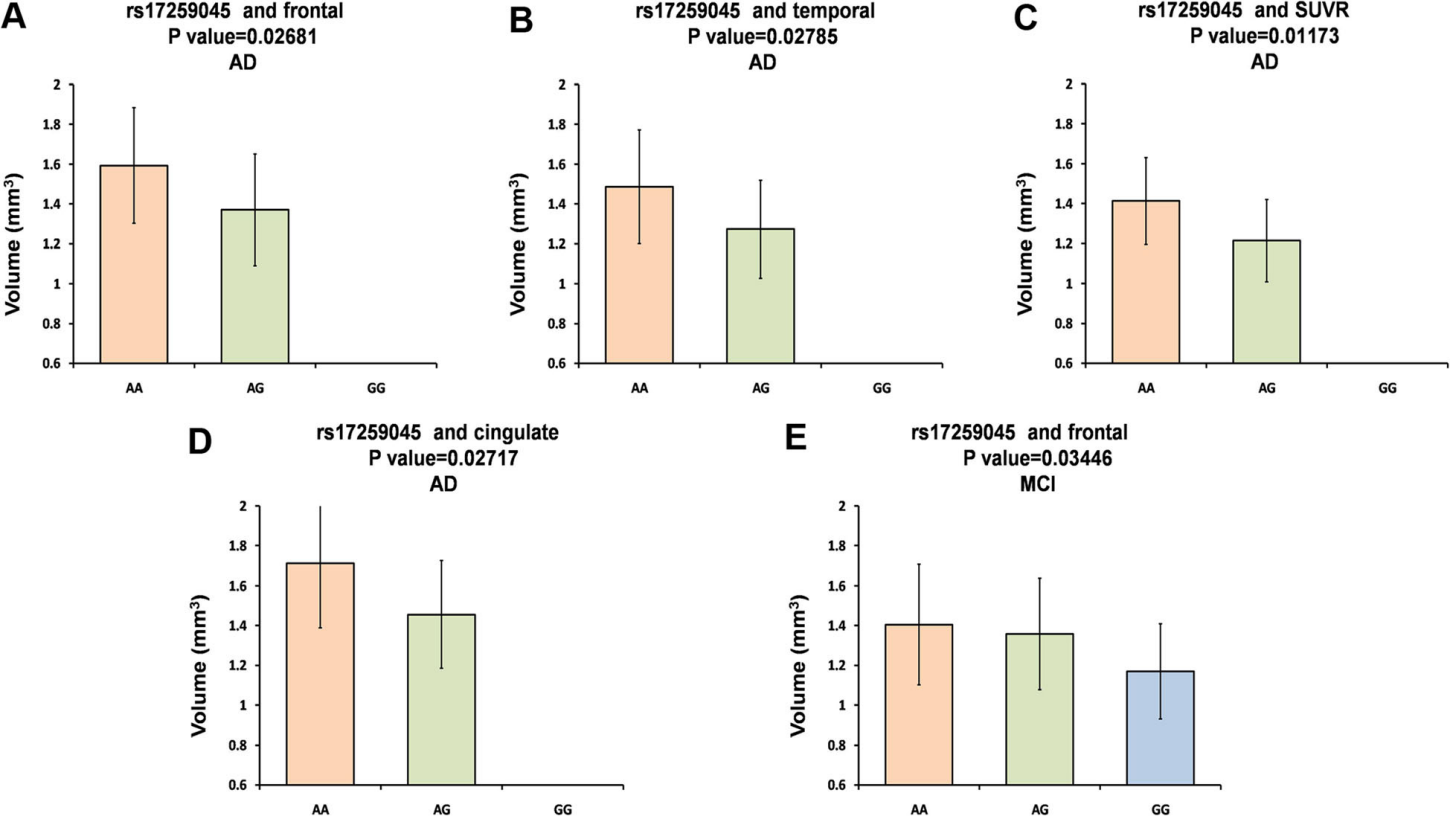
The analysis about the associations of the variants and Aβ accumulation. The rs17259045 decreased Aβ accumulation in frontal **a**, temporal **b**, SUVR **c**, and cingulate **d** of AD patients

Then, we conducted further analyses about the associations of the variants and Aβ accumulation in AD and NC population. As shown in Table 3 and Fig. 1, rs17259045 may decrease Aβ accumulation of AD patients in frontal (AA: mean ± SD, 1.593 ± 0.2906, *N* = 37; AG: mean ± SD, 1.37 ± 0.2805, *N* = 9; *P* = 0.02681), temporal (AA: mean ± SD, 1.486 ± 0.2857, N = 37; AG: mean ± SD, 1.273 ± 0.2458, N = 9; *P* = 0.02785), SUVR (AA: mean ± SD, 1.413 ± 0.2178, N = 37; AG: mean ± SD, 1.214 ± 0.2062, N = 9; *P* = 0.01173), and cingulated (AA: mean ± SD, 1.711 ± 0.3232, N = 37; AG: mean ± SD, 1.455 ± 0.2704, N = 9; *P* = 0.02717).

**Table 3:** Stratified of positive results in AD group and NC group

Kinds	SNPs	Regions	Group	Value (mean ± SD)						β	***P*** value
				MM		Mm		mm			
				(mean ± SD)	***N***	(mean ± SD)	N	(mean ± SD)	***N***		
AV45-PET	rs10494884	temporal	AD	1.387 ± 0.2783	17	1.415 ± 0.3144	17	1.566 ± 0.2483	12	0.06873	0.119
		temporal	NC	1.201 ± 0.2014	44	1.219 ± 0.2556	85	1.333 ± 0.2917	24	0.05121	0.08755
		frontal	AD	1.489 ± 0.2873	17	1.501 ± 0.3219	17	1.701 ± 0.2474	12	0.08014	0.08302
		frontal	NC	1.259 ± 0.2147	44	1.295 ± 0.2868	85	1.392 ± 0.2961	24	0.04313	0.1718
		SUVR	AD	1.33 ± 0.2193	17	1.332 ± 0.2515	17	1.494 ± 0.17	12	0.06683	0.06938
		SUVR	NC	1.103 ± 0.1775	44	1.125 ± 0.2015	85	1.201 ± 0.2176	24	0.03969	0.09432
	rs11118322	temporal	AD	1.387 ± 0.2783	17	1.415 ± 0.3144	17	1.566 ± 0.2483	12	0.06873	0.119
		temporal	NC	1.201 ± 0.2014	44	1.219 ± 0.2556	85	1.333 ± 0.2917	24	0.05121	0.08755
		frontal	AD	1.489 ± 0.2873	17	1.501 ± 0.3219	17	1.701 ± 0.2474	12	0.08014	0.08302
		frontal	NC	1.259 ± 0.2147	44	1.295 ± 0.2868	85	1.392 ± 0.2961	24	0.04313	0.1718
	rs1323721	temporal	AD	1.387 ± 0.2783	17	1.415 ± 0.3144	17	1.566 ± 0.2483	12	0.06873	0.119
		temporal	NC	1.2 ± 0.2035	43	1.219 ± 0.2541	86	1.333 ± 0.2917	24	0.05124	0.08938
		frontal	AD	1.489 ± 0.2873	17	1.501 ± .3219	17	1.701 ± 0.2474	12	0.08014	0.08302
		frontal	NC	1.257 ± 0.2169	43	1.295 ± 0.2851	86	1.392 ± 0.2961	24	0.04191	0.1878
	rs17259045	frontal	AD	**1.593 ± 0.2906**	**37**	**1.37 ± 0.2805**	**9**	**0**	**0**	**−0.1974**	**0.02681**
		frontal	NC	1.312 ± 0.2783	120	1.24 ± 0.2391	30	1.358 ± 0.3367	2	−0.04546	0.3264
		temporal	AD	**1.486 ± 0.2857**	**37**	**1.273 ± 0.2458**	**9**	**0**	**0**	**−0.1864**	**0.02785**
		temporal	NC	1.242 ± 0.2563	120	1.186 ± 0.2276	30	1.168 ± 0.2109	2	−0.05859	0.1847
		SUVR	AD	**1.413 ± 0.2178**	**37**	**1.214 ± 0.2062**	**9**	**0**	**0**	**−0.1775**	**0.01173**
		SUVR	NC	1.14 ± 0.2074	120	1.085 ± 0.1543	30	1.169 ± 0.2835	2	−0.04196	0.2297
		cingulate	AD	**1.711 ± 0.3232**	**37**	**1.455 ± 0.2704**	**9**	**0**	**0**	**−0.2262**	**0.02717**
		cingulate	NC	1.431 ± 0.3073	120	1.351 ± 0.2576	30	1.482 ± 0.2336	2	−0.06105	0.2494
		parietal	AD	1.604 ± 0.2992	37	1.369 ± 0.2273	9	0	0	−0.2085	0.01895
		parietal	NC	1.33 ± 0.29	120	1.251 ± 0.2344	30	1.341 ± 0.353	2	−0.06408	0.1983
	rs41308433	temporal	AD	1.5 ± 0.3029	30	1.33 ± 0.2385	15	1.498 ± 0	1	−0.08598	0.1943
			NC	1.252 ± 0.2708	105	1.188 ± 0.1949	45	1.151 ± 0.1519	3	−0.05517	0.1494
CSF Aβ_42_	rs12567945	CSF Aβ_42_	AD	139.6 ± 41.82	34	140.3 ± 34.01	5	233.9 ± 0	1	14.13	0.3726
			NC	**191.6 ± 53.29**	**116**	**219.7 ± 45.29**	**16**	**0**	**0**	**28.66**	**0.02589**

*AD* alzheimer’s disease, *NC* normal controls, *SNPs* single nucleotide polymorphisms, *SD* standard deviations, *SUVR* standardized uptake values ratios

### The effects of CR1 genetic variants on CSF Aβ_42_ biomarkers

**Fig. 2:**
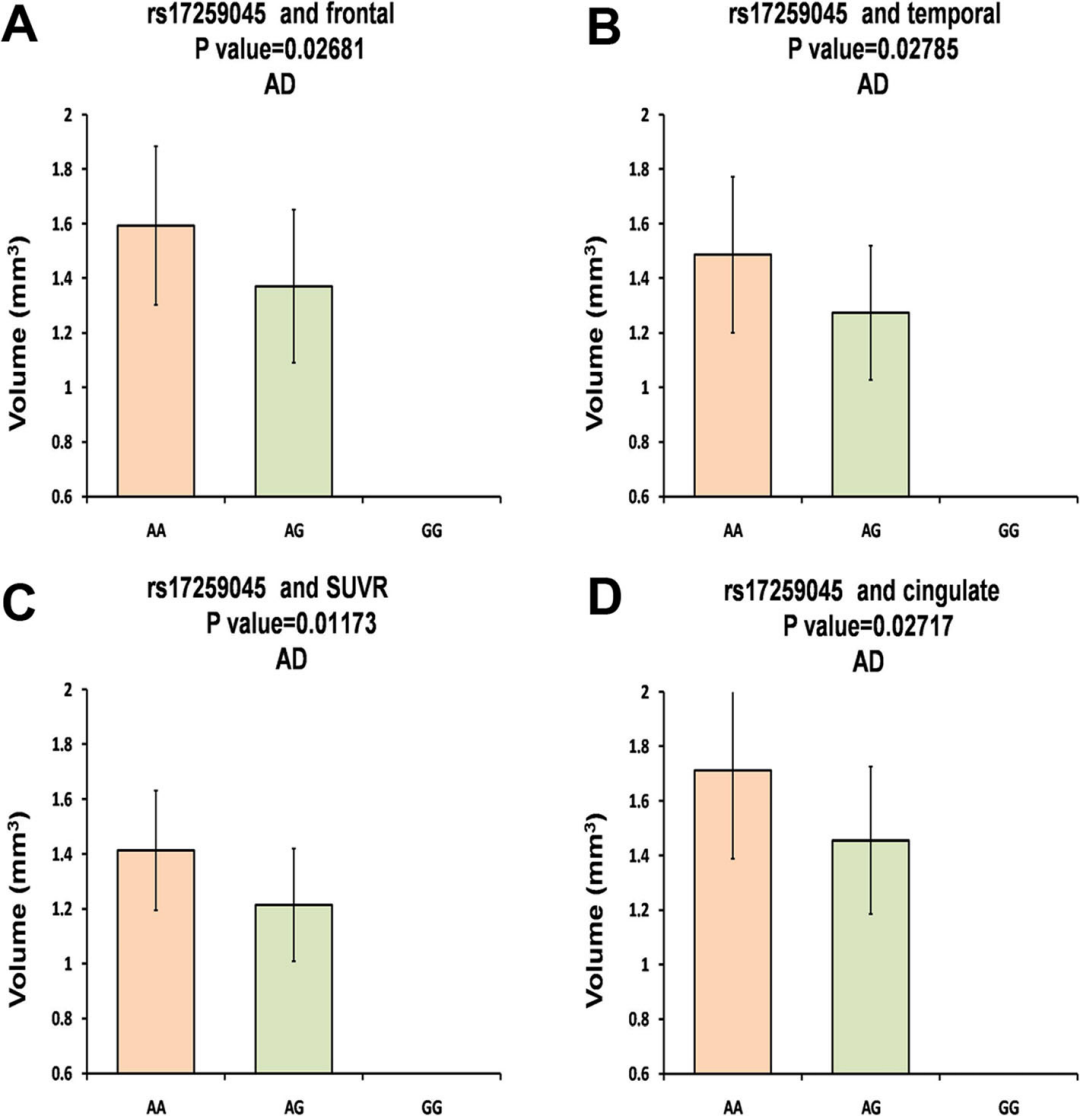
Further analyses about the associations of the rs12567945 and CSF Aβ_42_ in NC population

Next, the correlations between *CR1* genetic variants and CSF Aβ_42_ biomarkers were determined. The results indicated that rs12567945 could observably increase CSF Aβ_42_ in NC population (TT: mean ± SD, 191.6 ± 53.29, *N* = 116; TC: mean ± SD, 219.7 ± 45.29, *N* = 16; *P* = 0.02589; Table 3 and Fig. 2), which was found in block 3.

## Discussion

In our study, we explored the relation between whole *CR1* genetic variants and Aβ metabolism biomarkers, and the results showed that five SNPs, including rs10494884, rs11118322, rs1323721, rs17259045 and rs41308433 could significantly alter Aβ accumulation in brain. In further analyses, the results suggested rs17259045 might decrease Aβ accumulation among AD patients. In addition, the genetic variants in rs12567945 would increase CSF Aβ_42_ in NC population.

As we all known, Aβ is one important pathological characteristic of AD [27], which may induce the activation of the classical complement pathway in AD brains [28, 29]. Moreover, *CR1* is a necessary component of complement system, and it has been reported to have a close connection with amyloid plaque burden during aging [30, 31]. More importantly, *CR1* genetic variants are found to link to intelligence decline, and may influence the eliminations of Aß plaques [30]. Hence, it is urgent to investigate whether *CR1* polymorphisms take part in the pathogenesis and development of LOAD. Actually, previous studies have revealed the association between *CR1* SNPs and amyloid plaque [30, 32–34], including the CSF Aβ levels [35–37]. However, the current studies only discuss the role of specific SNPs (rs6656401, rs3818361, rs670173, and rs1408077) in Aβ metabolism. Briefly, rs6656401 and rs3818361, within the CR1 gene, have association with LOAD susceptibility in Caucasians [17], which are found to be in moderate LD (D′ = 0.824, r^2^ = 0.328) [38]. Specially, rs3818361 is found to be in block 1 [37]. In our study, the results firstly revealed that rs17259045 could reduce the level of Aβ accumulation among AD patients, respectively; moreover, rs12567945 could increase CSF Aβ_42_ in NC population. In fact, rs17259045 was in the missense of *CR1* gene, and rs12567945 located in the intron variant of *CR1* gene. We speculated the genetic variants in the two SNPs might modulate the level of *CR1*, influence the activation of complement system, and finally alter the Aβ metabolism in the clearance of Aβin the brain. Taken together, these results indicated that the detection of variants in *CR1* gene may be useful to diagnose AD timely, and it may be a useful method to treat AD via altering *CR1* level.

**Fig. 3:**
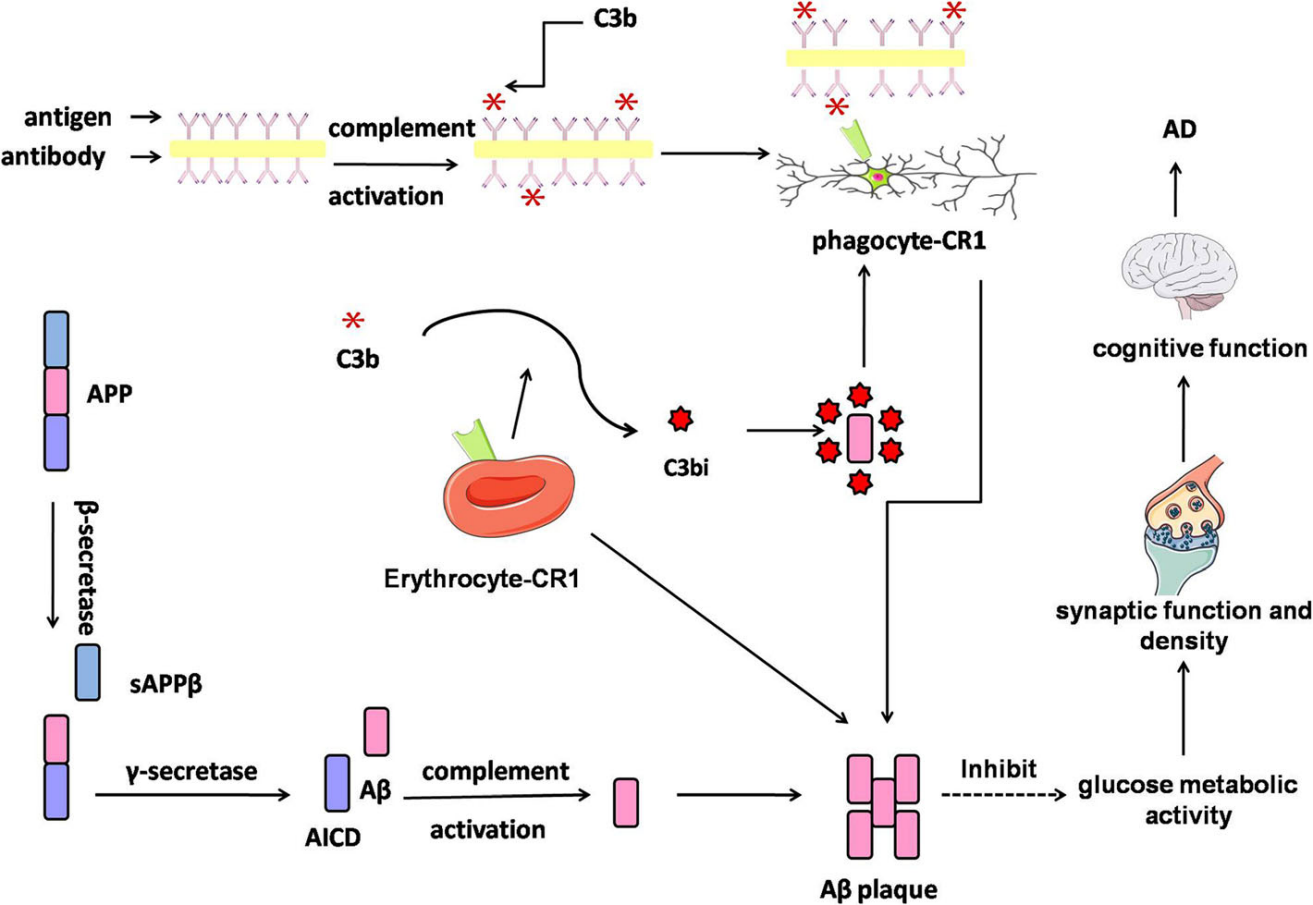
The possible pathway related to *CR1* in Aβ metabolism

Our previous study had reported that several volume (entorhinal, middle temporal, posterior cingulate, precuneus, parahippocampal), volume of subcortical (amygdale and hippocampus) and CA1 (the most associated area with the AD-specific amnenstic syndrome in hippocampus) were significantly related to AD [39]. However, our study failed to find the association between the genetic variants of *CR1* (rs17259045 and rs12567945) and the above regions of interest via using ADNI data. As well know, one characteristic feature of synaptic function and density is cerebral glucose metabolic activity. Moreover, the change of glucose metabolic activity in specific brain regions could be valued via FDG PET [40]. Our study indicated that AD patents with genetic variants in rs17259045 might have more level of glucose metabolic activity in right angular (*P* = 0.03278). Hence, we hypothesized that genetic variants in *CR1* might influence cognitive function (Fig. 3), through regulating CSF Aβ level, changing Aβ accumulations in brains, influencing the glucose metabolic activity, as well as altering the synaptic function and density.

## Conclusion

In summary, our study found five SNPs (rs10494884, rs11118322, rs1323721, rs17259045 and rs41308433) were significantly linked to Aβ accumulation in brain. In further analyses of positive results, rs17259045 was found to decrease Aβ accumulation among AD patients. In addition, our study indicated genetic variants in rs12567945 would increase CSF Aβ_42_ in NC population. Taken together, our study revealed some novel SNPs in *CR1* which might be involved in AD development through regulating the Aβ pathology. However, several limitations still exist in this study. Firstly, the numbers of included samples were relative small. Secondly, our study was explored only in Caucasians. Hence, further study with larger samples and different ethnicities is still necessary.

## Supplementary information

**Additional file 1.**

**Additional file 2.**

## Data Availability

Not applicable. This study was only the primary research, and further study has been in progress.

## Abbreviations

AD: Alzheimer’s disease
ADNI: Alzheimer’s Disease Neuroimaging Initiative
ADRDA: Alzheimer’s Disease and Related Disorders Association
Aβ: amyloid-β
CSF: Cerebrospinal fluid
FDR: False discovery rate
MAF: Minor allele frequency
MCI: Mild cognitive impairment
MMSE: Mini-mental state examination
NC: Normal controls
NINCDS: Neurological and Communicative Disorders
SNP: Single nucleotide polymorphism
SUVR: Standardized uptake values ratios
