# Supplementary figures and images for: Impacts of CR1 genetic variants on cerebrospinal fluid and neuroimaging biomarkers in alzheimer’s disease
Source: BMC Med Genet. 2020 Sep 12;21:181. doi: 10.1186/s12881-020-01114-x (PMC7488421; doi:10.1186/s12881-020-01114-x)

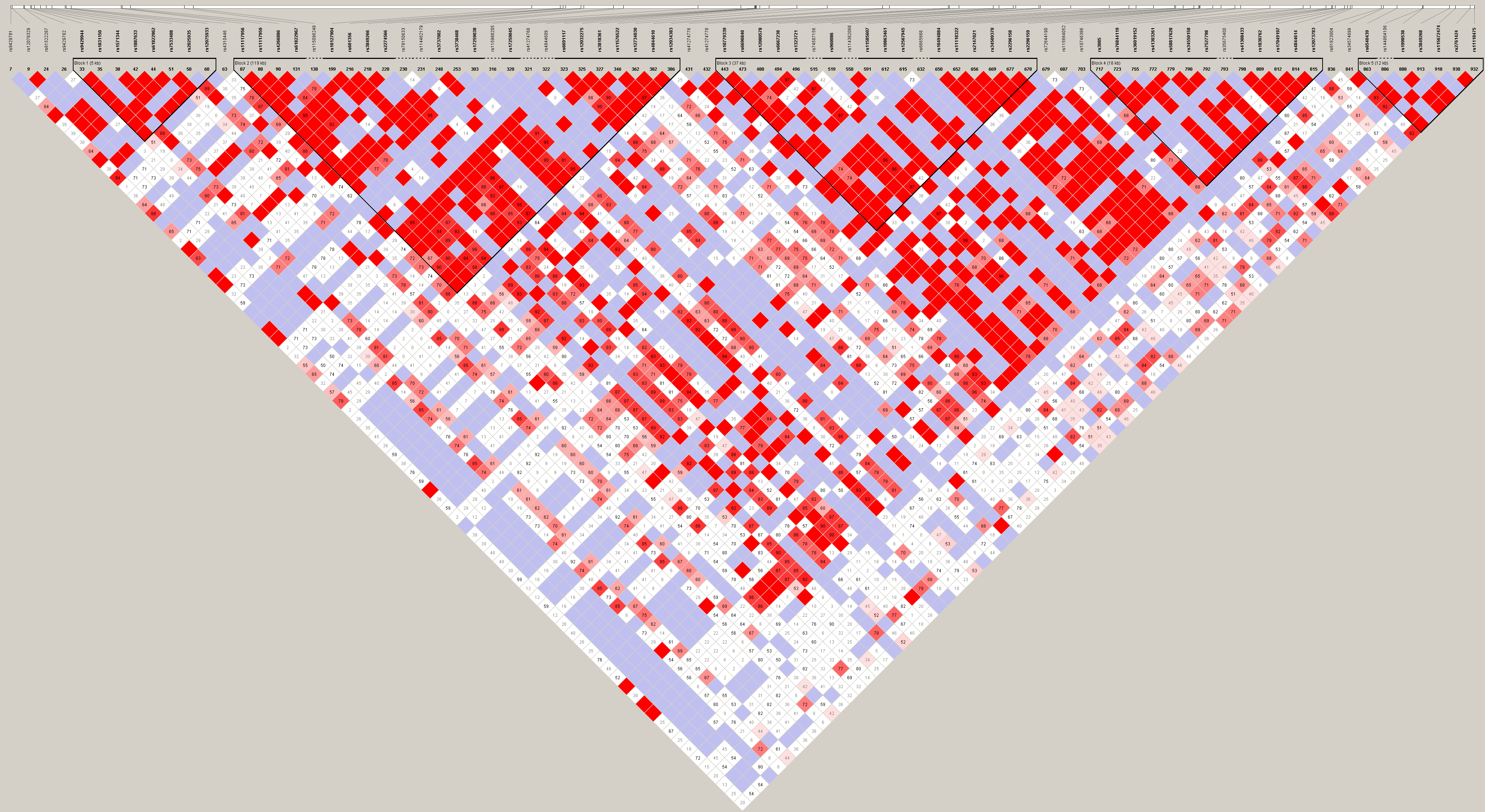

Supplement: Supplementary file 1 — Additional file 1. [file 12881_2020_1114_MOESM1_ESM.tif]
