# Supplementary material for: Impacts of CR1 genetic variants on cerebrospinal fluid and neuroimaging biomarkers in alzheimer’s disease
Source: BMC Med Genet. 2020 Sep 12;21:181. doi: 10.1186/s12881-020-01114-x (PMC7488421; doi:10.1186/s12881-020-01114-x)
Supplement: Supplementary file 2 — Additional file 2. [file 12881_2020_1114_MOESM2_ESM.docx]

**Supplementary table 1. The details of included SNPs in *CR1***

| **SNPs** | **Major allele**  **(M)** | **Minor allele**  **(m)** | **MAF**  **(1000)** | **Functional Consequence** | **Position**  **(Chromosome)** | **CSF**  **MAF** | **MM** | **Mm** | **mm** | **CSF**  **H-W**  **(*p* value)** | **AV-45**  **MAF** | **MM** | **Mm** | **mm** | **AV-45 H-W**  **(*p* value)** |
| --- | --- | --- | --- | --- | --- | --- | --- | --- | --- | --- | --- | --- | --- | --- | --- |
| rs11118475 | G | A | 0.42 | intron variant | 1:207721071 | 0.067797 | 412 | 56 | 4 | 0.18248 | 0.494444 | 133 | 280 | 127 | 0.38779 |
| rs9429781 | T | G | 0.19 | upstream gene variant | 1:207493877 | 0.230932 | 275 | 176 | 21 | 0.27963 | 0.230556 | 316 | 199 | 25 | 0.36889 |
| rs12078329 | C | T | 0.08 | upstream gene variant | 1:207667497 | 0.041226 | 435 | 37 | 1 | 0.81957 | 0.041667 | 497 | 41 | 2 | 0.25219 |
| rs61522287 | G | A | 0.04 | upstream variant 2KB | 1:207669248 | 0.037155 | 436 | 35 | 0 | 0.40233 | 0.035316 | 500 | 38 | 0 | 0.39581 |
| rs9429782 | G | T | 0.09 | upstream variant 2KB | 1:207669355 | 0.011677 | 460 | 11 | 0 | 0.79763 | 0.010204 | 528 | 11 | 0 | 0.81084 |
| rs9429944 | A | T | 0.24 | intron variant | 1:207670307 | 0.265393 | 251 | 190 | 30 | 0.45315 | 0.263451 | 290 | 214 | 35 | 0.59267 |
| rs1831150 |  | G | 0.14 | intron variant | 1:207670438 | 0.283898 | 236 | 204 | 32 | 0.17130 | 0.29537 | 264 | 233 | 43 | 0.39525 |
| rs1571344 | T | C | 0.15 | intron variant | 1:207670928 | 0.138004 | 348 | 116 | 7 | 0.44536 | 0.140074 | 397 | 133 | 9 | 0.57315 |
| rs1887633 | C | A | 0.15 | intron variant | 1:207498504 | 0.137712 | 349 | 116 | 7 | 0.44944 | 0.139815 | 398 | 133 | 9 | 0.57769 |
| rs61822962 | G | T | 0.15 | intron variant | 1:207499374 | 0.137712 | 349 | 116 | 7 | 0.44944 | 0.139815 | 398 | 133 | 9 | 0.57769 |
| rs7533408 | C | T | 0.2444 | intron variant | 1:207500286 | 0.268008 | 249 | 193 | 30 | 0.35979 | 0.265741 | 288 | 217 | 35 | 0.48948 |
| rs2025935 | C | T | 0.1861 | intron variant | 1:207502310 | 0.288136 | 232 | 208 | 32 | 0.10681 | 0.300926 | 259 | 237 | 44 | 0.57769 |
| rs12075933 | T | G | 0.1522 | intron variant | 1:207502508 | 0.138771 | 348 | 117 | 7 | 0.42097 | 0.140741 | 397 | 134 | 9 | 0.54610 |
| rs4310446 | T | C | 0.1046 | intron variant | 1:207503259 | 0.186837 | 309 | 148 | 14 | 0.45899 | 0.184601 | 355 | 169 | 15 | 0.33519 |
| rs11117956 | T | G | 0.3377 | intron variant | 1:207511014 | 0.214744 | 291 | 153 | 24 | 0.50739 | 0.202425 | 344 | 167 | 25 | 0.41683 |
| rs11117959 | A | G | 0.275 | intron variant | 1:207511174 | 0.195975 | 305 | 149 | 18 | 0.97026 | 0.184259 | 360 | 161 | 19 | 0.84873 |
| rs4266886 | C | T | 0.1426 | intron variant | 1:207512441 | 0.151442 | 302 | 102 | 12 | 0.34813 | 0.152542 | 342 | 116 | 14 | 0.28273 |
| rs61822967 | G | A | 0.2662 | intron variant | 1:207520820 | 0.304025 | 226 | 205 | 41 | 0.56759 | 0.305556 | 260 | 230 | 50 | 0.93266 |
| rs115096248 | C | G | 0.0036 | intron variant | 1:207522243 | 0.018008 | 455 | 17 | 0 | 0.69032 | 0.016667 | 522 | 18 | 0 | 0.69368 |
| rs10127904 | T | G | 0.1206 | intron variant | 1:207574762 | 0.304255 | 222 | 210 | 38 | 0.23002 | 0.310056 | 250 | 241 | 46 | 0.25656 |
| rs601356 | T | G | 0.4906 | intron variant | 1:207579205 | 0.218684 | 291 | 154 | 26 | 0.34861 | 0.20872 | 342 | 169 | 28 | 0.23857 |
| rs3849266 | C | T | 0.2478 | intron variant | 1:207579645 | 0.279661 | 242 | 196 | 34 | 0.50535 | 0.282407 | 277 | 221 | 42 | 0.82073 |
| rs2274566 | T | C | 0.3828 | intron variant | 1:207580000 | 0.404255 | 169 | 222 | 79 | 0.67468 | 0.395911 | 196 | 258 | 84 | 0.95271 |
| rs78150633 | G | A | 0.0036 | intron variant | 1:207582391 | 0.016985 | 455 | 16 | 0 | 0.70767 | 0.01577 | 522 | 17 | 0 | 0.70990 |
| rs114462179 | C | T | 0.0136 | intron variant | 1:207582842 | 0.023305 | 451 | 20 | 1 | 0.13264 | 0.022222 | 517 | 22 | 1 | 0.14640 |
| rs3737002 | C | T | 0.2488 | missense | 1:207587428 | 0.27766 | 242 | 195 | 33 | 0.45694 | 0.27974 | 278 | 219 | 41 | 0.81378 |
| rs3738468 | C | T | 0.2776 | intron variant | 1:207589013 | 0.192797 | 309 | 144 | 19 | 0.66684 | 0.182407 | 363 | 157 | 20 | 0.55748 |
| rs17259038 | A | G | 0.1208 | intron variant | 1:207600557 | 0.306794 | 222 | 209 | 40 | 0.34798 | 0.312616 | 250 | 241 | 48 | 0.34864 |
| rs115988205 | C | A | 0.0030 | intron variant | 1:207607240 | 0.010593 | 462 | 10 | 0 | 0.81607 | 0.010185 | 529 | 11 | 0 | 0.81101 |
| rs17259045 | A | G | 0.0571 | missense | 1:207609362 | 0.08811 | 391 | 77 | 3 | 0.70652 | 0.091837 | 445 | 89 | 5 | 0.81459 |
| rs41274768 | G | A | 0.0132 | missense | 1:207609424 | 0.020127 | 454 | 17 | 1 | 0.05908 | 0.019444 | 520 | 19 | 1 | 0.07246 |
| rs4844609 | T | A | 0.0050 | missense | 1:207609571 | 0.028723 | 444 | 25 | 1 | 0.31142 | 0.031599 | 504 | 34 | 0 | 0.44915 |
| rs6691117 | A | G | 0.4934 | missense | 1:207609586 | 0.21822 | 293 | 152 | 27 | 0.22231 | 0.208333 | 344 | 167 | 29 | 0.14668 |
| rs12032275 | C | T | 0.2718 | intron variant | 1:207610162 | 0.190678 | 311 | 142 | 19 | 0.58334 | 0.180556 | 365 | 155 | 20 | 0.48591 |
| rs3818361 | C | T | 0.2486 | intron variant | 1:207611623 | 0.189619 | 307 | 151 | 14 | 0.37352 | 0.191667 | 348 | 177 | 15 | 0.17906 |
| rs11576522 | G | A | 0.3998 | intron variant | 1:207615924 | 0.337384 | 178 | 226 | 68 | 0.78386 | 0.376852 | 205 | 263 | 72 | 0.39017 |
| rs12734030 | C | T | 0.2584 | intron variant | 1:207620619 | 0.174788 | 322 | 135 | 15 | 0.85316 | 0.165741 | 377 | 147 | 16 | 0.71664 |
| rs4844610 | C | A | 0.0647 | intron variant | 1:207629207 | 0.18259 | 312 | 146 | 13 | 0.40405 | 0.184601 | 354 | 171 | 14 | 0.21135 |
| rs12034383 | A | G | 0.4060 | intron variant | 1:207630250 | 0.403602 | 167 | 229 | 76 | 0.86545 | 0.396296 | 193 | 266 | 81 | 0.49345 |
| rs41274776 | A | G | 0.1196 | utr variant 3 prime | 1:207639446 | 0.051613 | 418 | 46 | 1 | 0.82108 | 0.051595 | 480 | 51 | 2 | 0.60696 |
| rs41274778 | C | T | 0.0399 | utr variant 3 prime | 1:207639613 | 0.039195 | 436 | 35 | 1 | 0.73688 | 0.037963 | 500 | 39 | 1 | 0.79386 |
| rs10779339 | C | T | 0.4173 | utr variant 3 prime | 1:207641106 | 0.476695 | 125 | 244 | 103 | 0.43224 | 0.486111 | 141 | 273 | 126 | 0.78228 |
| rs6696840 | T | C | 0.4601 | intron variant | 1:207646384 | 0.48411 | 120 | 247 | 105 | 0.30038 | 0.475926 | 145 | 276 | 119 | 0.56760 |
| rs12080578 | A | G | 0.0905 | intron variant | 1:207647267 | 0.04034 | 434 | 36 | 1 | 0.78103 | 0.038961 | 498 | 40 | 1 | 0.83433 |
| rs6667238 | G | A | 0.3700 | intron variant | 1:207649511 | 0.425847 | 147 | 248 | 77 | 0.10564 | 0.417593 | 176 | 277 | 87 | 0.20475 |
| rs1323721 | A | G | 0.4599 | intron variant | 1:207649895 | 0.466102 | 127 | 250 | 95 | 0.16299 | 0.456481 | 153 | 281 | 106 | 0.25791 |
| rs74587156 | T | G | 0.0054 | intron variant | 1:207653478 | 0.013771 | 459 | 13 | 0 | 0.76161 | 0.014815 | 524 | 16 | 0 | 0.72676 |
| rs960086 | A | G | 0.3716 | intron variant | 1:207653724 | 0.425847 | 147 | 248 | 77 | 0.10564 | 0.417593 | 176 | 277 | 87 | 0.20475 |
| rs114382068 | C | T | 0.0092 | intron variant | 1:207658449 | 0.021368 | 449 | 18 | 1 | 0.08217 | 0.020561 | 514 | 20 | 1 | 0.09665 |
| rs11585607 | A | G | 0.0397 | intron variant | 1:207664395 | 0.039278 | 435 | 35 | 1 | 0.73854 | 0.038033 | 499 | 39 | 1 | 0.79535 |
| rs10863461 | G | A | 0.3860 | intron variant | 1:207667431 | 0.420551 | 150 | 247 | 75 | 0.10924 | 0.412963 | 179 | 276 | 85 | 0.20814 |
| rs12567945 | T | C | 0.1238 | intron variant | 1:207668589 | 0.057203 | 419 | 52 | 1 | 0.64214 | 0.056481 | 481 | 57 | 2 | 0.82281 |
| rs6665668 | C | T | 0.0156 | intron variant | 1:207671274 | 0.03397 | 439 | 32 | 0 | 0.44536 | 0.032468 | 504 | 35 | 0 | 0.43594 |
| rs10494884 | G | A | 0.3994 | intron variant | 1:207674531 | 0.462845 | 129 | 248 | 94 | 0.20095 | 0.451763 | 157 | 277 | 105 | 0.38415 |
| rs11118322 | T | C | 0.3994 | intron variant | 1:207674706 | 0.463983 | 129 | 248 | 95 | 0.22104 | 0.452778 | 157 | 277 | 106 | 0.41391 |
| rs2147021 | A | G | 0.1070 | intron variant | 1:207675869 | 0.26589 | 253 | 187 | 32 | 0.74680 | 0.272222 | 287 | 212 | 41 | 0.83087 |
| rs34509370 | C | T | 0.1068 | synonymous codon | 1:207677534 | 0.264831 | 254 | 186 | 32 | 0.78140 | 0.271296 | 288 | 211 | 41 | 0.78470 |
| rs2296158 | G | A | 0.4734 | missense | 1:207678266 | 0.463357 | 122 | 210 | 91 | 0.97161 | 0.471992 | 137 | 235 | 110 | 0.63180 |
| rs2296159 | G | A | 0.3315 | intron variant | 1:207678415 | 0.420213 | 152 | 241 | 77 | 0.25660 | 0.410781 | 183 | 268 | 87 | 0.50047 |
| rs72644190 | G | T | 0.1609 | intron variant | 1:207678461 | 0.09448 | 384 | 85 | 2 | 0.23514 | 0.092764 | 442 | 94 | 3 | 0.40178 |
| rs115664052 | G | A | 0.0052 | intron variant | 1:207680468 | 0.013771 | 459 | 13 | 0 | 0.76161 | 0.015741 | 523 | 17 | 0 | 0.71017 |
| rs10746386 | C | T | 0.3355 | intron variant | 1:207681750 | 0.419913 | 152 | 232 | 78 | 0.50832 | 0.410208 | 183 | 258 | 88 | 0.85525 |
| rs3085 | A | G | 0.1611 | missense | 1:207683909 | 0.09428 | 385 | 85 | 2 | 0.23664 | 0.092593 | 443 | 94 | 3 | 0.40390 |
| rs76844119 | G | A | 0.0397 | intron variant | 1:207684828 | 0.040254 | 435 | 36 | 1 | 0.77934 | 0.038889 | 499 | 40 | 1 | 0.83283 |
| rs36019152 | T | A | 0.1064 | intron variant | 1:207689353 | 0.264831 | 254 | 186 | 32 | 0.79413 | 0.271296 | 288 | 211 | 41 | 0.78470 |
| rs41303261 | G | A | 0.0393 | missense | 1:207694509 | 0.038217 | 435 | 36 | 0 | 0.38849 | 0.037106 | 499 | 40 | 0 | 0.37097 |
| rs58817628 | C | G | 0.2923 | intron variant | 1:207695627 | 0.364119 | 184 | 231 | 56 | 0.19953 | 0.368275 | 211 | 259 | 69 | 0.44753 |
| rs34550158 | C | T | 0.0224 | intron variant | 1:207698062 | 0.075532 | 401 | 67 | 2 | 0.65263 | 0.076208 | 459 | 76 | 3 | 0.93921 |
| rs7527798 | T | C | 0.1114 | intron variant | 1:207698945 | 0.263326 | 254 | 183 | 32 | 0.90134 | 0.268224 | 288 | 207 | 40 | 0.73947 |
| rs35675400 | C | T | 0.0032 | intron variant | 1:207699060 | 0.020213 | 451 | 19 | 0 | 0.65470 | 0.021375 | 515 | 23 | 0 | 0.61241 |
| rs41308433 | A | C | 0.1619 | intron variant | 1:207699490 | 0.154989 | 335 | 126 | 10 | 0.64381 | 0.160482 | 380 | 145 | 14 | 0.96984 |
| rs1830762 | C | T | 0.2841 | intron variant | 1:207700927 | 0.324094 | 209 | 216 | 44 | 0.26732 | 0.328067 | 240 | 243 | 55 | 0.57009 |
| rs17049197 | G | A | 0.2620 | intron variant | 1:207701359 | 0.225446 | 265 | 164 | 19 | 0.30771 | 0.230545 | 301 | 189 | 24 | 0.40915 |
| rs4844614 | G | T | 0.3215 | intron variant | 1:207701830 | 0.242585 | 266 | 183 | 23 | 0.23152 | 0.246753 | 302 | 208 | 29 | 0.37625 |
| rs12073783 | A | G | 0.3181 | intron variant | 1:207702086 | 0.364407 | 184 | 232 | 56 | 0.18447 | 0.367593 | 211 | 261 | 68 | 0.35784 |
| rs61823004 | T | C | 0.0042 | intron variant | 1:207706631 | 0.018008 | 455 | 17 | 0 | 0.69032 | 0.018519 | 520 | 20 | 0 | 0.66106 |
| rs34574699 | A | G | 0.1124 | intron variant | 1:207706983 | 0.157113 | 330 | 134 | 7 | 0.10746 | 0.154917 | 381 | 149 | 9 | 0.19538 |
| rs6540439 | C | T | 0.1394 | intron variant | 1:207709056 | 0.271277 | 245 | 195 | 30 | 0.28440 | 0.27881 | 275 | 226 | 37 | 0.30123 |
| rs144864106 | A | G | 0.0224 | intron variant | 1:207713079 | 0.038217 | 435 | 36 | 0 | 0.38849 | 0.036178 | 500 | 39 | 0 | 0.38351 |
| rs1998538 | G | A | 0.3039 | intron variant | 1:207713142 | 0.433263 | 146 | 243 | 83 | 0.29364 | 0.438889 | 163 | 280 | 97 | 0.22016 |
| rs3849268 | A | G | 0.4205 | intron variant | 1:207717223 | 0.492585 | 119 | 241 | 112 | 0.64181 | 0.494444 | 133 | 280 | 127 | 0.38779 |
| rs115672474 | C | T | 0.0296 | intron variant | 1:207718166 | 0.058263 | 418 | 53 | 1 | 0.61342 | 0.054731 | 481 | 57 | 1 | 0.61757 |
| rs2761424 | C | T | 0.1591 | intron variant | 1:207720437 | 0.286938 | 234 | 198 | 35 | 0.43527 | 0.294944 | 262 | 229 | 43 | 0.47233 |
